# Supplementary material for: Adaptation and validation of the Physical Activity Questionnaire for Older Children (PAQ-C) among Czech children
Source: PLoS One. 2021 Jan 12;16(1):e0245256. doi: 10.1371/journal.pone.0245256 (PMC7802927; doi:10.1371/journal.pone.0245256)
Supplement: S1 File — (DOCX) [file pone.0245256.s002.docx]

| **Physical Activity Questionnaire for Older Children (PAQ-C)** | |
| --- | --- |
| **English** | **Czech** |
| Physical Activity Questionnaire for Older Children (PAQ-C) | Dotazník pohybové aktivity dětí (PAQ-C/cz) |
| 1. Physical activity in your spare time: Have you done any of the following activities in the past 7 days (last week)? If yes, how many times? (Mark only one circle per row.)  \|  \| No \| 1-2 \| 3-4 \| 5-6 \| 7 times or more \| \| --- \| --- \| --- \| --- \| --- \| --- \| \| Skipping .............................. \| օ \| օ \| օ \| օ \| օ \| \| Rowing/canoeing .................. \| օ \| օ \| օ \| օ \| օ \| \| In-line skating ....................... \| օ \| օ \| օ \| օ \| օ \| \| Tag .......................................... \| օ \| օ \| օ \| օ \| օ \| \| Walking for exercise ............... \| օ \| օ \| օ \| օ \| օ \| \| Bicycling ................................ \| օ \| օ \| օ \| օ \| օ \| \| Jogging or running .................. \| օ \| օ \| օ \| օ \| օ \| \| Aerobics ................................. \| օ \| օ \| օ \| օ \| օ \| \| Swimming .............................. \| օ \| օ \| օ \| օ \| օ \| \| Baseball, softball .................... \| օ \| օ \| օ \| օ \| օ \| \| Dance ..................................... \| օ \| օ \| օ \| օ \| օ \| \| Football .................................. \| օ \| օ \| օ \| օ \| օ \| \| Badminton .............................. \| օ \| օ \| օ \| օ \| օ \| \| Skateboarding ......................... \| օ \| օ \| օ \| օ \| օ \| \| Soccer ...................................... \| օ \| օ \| օ \| օ \| օ \| \| Street hockey .......................... \| օ \| օ \| օ \| օ \| օ \| \| Volleyball ............................... \| օ \| օ \| օ \| օ \| օ \| \| Floor hockey ........................... \| օ \| օ \| օ \| օ \| օ \| \| Basketball ................................ \| օ \| օ \| օ \| օ \| օ \| \| Ice skating ............................... \| օ \| օ \| օ \| օ \| օ \| \| Cross-country skiing ............... \| օ \| օ \| օ \| օ \| օ \| \| Ice hockey/ringette .................. \| օ \| օ \| օ \| օ \| օ \| \| Other: \| օ \| օ \| օ \| օ \| օ \| \| _________________ ...... \| օ \| օ \| օ \| օ \| օ \| \| _________________ ...... \| օ \| օ \| օ \| օ \| օ \| | 1. V posledních 7 dnech: Kterým aktivitám ses věnoval nepřetržitě alespoň půl hodiny?   \| KROK 1 \| \| KROK 2 \| \| \| \| \| --- \| --- \| --- \| --- \| --- \| --- \| \| ANO \|  \| 1–2krát \| 3–4krát \| 5–6krát \| 7krát  a více \| \| օ \| Atletika (běhání, skákání, házení) \| օ \| օ \| օ \| օ \| \| օ \| Basketbal \| օ \| օ \| օ \| օ \| \| օ \| Bojové sporty (judo, karate a jiné) \| օ \| օ \| օ \| օ \| \| օ \| Florbal \| օ \| օ \| օ \| օ \| \| օ \| Fotbal \| օ \| օ \| օ \| օ \| \| օ \| Gymnastika \| օ \| օ \| օ \| օ \| \| օ \| Házená, vybíjená \| օ \| օ \| օ \| օ \| \| օ \| In-line bruslení, bruslení na ledě \| օ \| օ \| օ \| օ \| \| օ \| Jízda na kole, koloběžce (ne e-kolo a e-koloběžka) \| օ \| օ \| օ \| օ \| \| օ \| Jízda na koni \| օ \| օ \| օ \| օ \| \| օ \| Lední hokej \| օ \| օ \| օ \| օ \| \| օ \| Parkour, street workout \| օ \| օ \| օ \| օ \| \| օ \| Plavání a jiné aktivity ve vodě \| օ \| օ \| օ \| օ \| \| օ \| Posilovací cvičení, fitness, jóga \| օ \| օ \| օ \| օ \| \| օ \| Lyžování, běžky, snowboarding \| օ \| օ \| օ \| օ \| \| օ \| Skateboarding, penny board \| օ \| օ \| օ \| օ \| \| օ \| Softball, baseball \| օ \| օ \| օ \| օ \| \| օ \| Tanec, aerobik, hip-hop, street-dance \| օ \| օ \| օ \| օ \| \| օ \| Tenis, squash, stolní tenis, soft-tenis, badminton \| օ \| օ \| օ \| օ \| \| օ \| Turistika, delší chůze \| օ \| օ \| օ \| օ \| \| օ \| Volejbal, beach volejbal \| օ \| օ \| օ \| օ \| \| օ \| Jiné \| օ \| օ \| օ \| օ \| |
|  | 2. V posledních 5 školních dnech: V kolika dnech ses ráno před školou věnoval nějakému sportu, hraní her nebo jiným pohybovým aktivitám, u kterých jsi byl velmi aktivní (hodně ses u nich zadýchal, zpotil a unavil)?  (Označ křížkem pouze jednu odpověď.)  V žádném dni ................................................... օ  V 1 dni ............................................................. օ  Ve 2 nebo 3 dnech ............................................ օ  Ve 4 dnech ........................................................ օ  V 5 dnech ......................................................... օ |
| 2. In the last 7 days, during your physical education (PE) classes, how often were you very active (playing hard, running, jumping, throwing)? (Check one only.)  I don’t do PE .................................................... օ  Hardly ever ....................................................... օ  Sometimes ........................................................ օ  Quite often ....................................................... օ  Always .............................................................. օ | 3. V posledních 5 školních dnech: Kolikrát jsi byl v tělocviku velmi aktivní? Velmi aktivní je intenzivní hraní, běhání, skákání, házení, plavání, u kterého jsi byl hodně zadýchaný a zpocený. (Označ křížkem pouze jednu odpověď.)  Neměl jsem tělocvik nebo jsem necvičil .......... օ  Málokdy ............................................................ օ  Občas ................................................................ օ  Docela často ...................................................... օ  Skoro pořád nebo pořád ................................... օ |
| 3. In the last 7 days, what did you do most of the time at recess? (Check one only.)  Sat down (talking, reading, doing schoolwork) ......օ  Stood around or walked around ............................օ  Ran or played a little bit ........................................օ  Ran around and played quite a bit .........................օ  Ran and played hard most of the time ....................օ | 4. V posledních 5 školních dnech: Co jsi dělal po většinu času o všech přestávkách ve škole?  Počítej zde i dobu mezi příchodem do školy a začátkem vyučování. (Označ křížkem pouze jednu odpověď.)  Seděl (povídal, četl, plnil školní povinnosti) .................................................................. օ  Postával jsem nebo se pomalu procházel ......................................................................... օ  Trochu jsem pobíhal nebo si hrál (bez výraznějšího zadýchání) ..................................... օ  Docela hodně jsem pobíhal nebo si hrál (zadýchal jsem se víc, než při běžné chůzi) .... օ  Po většinu času jsem intenzivně běhal nebo si hrál (hodně jsem se zadýchal a zpotil) ... օ |
| 4. In the last 7 days, what did you normally do at lunch (besides eating lunch)?  (Check one only.)  Sat down (talking, reading, doing schoolwork) ... օ  Stood around or walked around ........................... օ  Ran or played a little bit ....................................... օ  Ran around and played quite a bit ....................... օ  Ran and played hard most of the time .................. օ |  |
| 5. In the last 7 days, on how many days right after school, did you do sports, dance, or play games in which you were very active? (Check one only.)  None .................................................................. օ  1 time last week .................................................. օ  2 or 3 times last week ........................................ օ  4 times last week ................................................ օ  5 times last week ................................................ օ | 5. V posledních 5 školních dnech: V kolika dnech ses hned po škole a odpoledne věnoval nějakému sportu, hraní her nebo jiným pohybovým aktivitám, u kterých jsi byl velmi aktivní (hodně ses zadýchal nebo zpotil)?  Jedná se o dobu mezi odchodem z budovy školy a přibližně 6 hodinou večer. (Označ křížkem pouze jednu odpověď.)  V žádném dni .................................................... օ  V 1 dni ............................................................. օ  Ve 2 nebo 3 dnech ............................................ օ  Ve 4 dnech ........................................................ օ  V 5 dnech ......................................................... օ |
| 6. In the last 7 days, on how many evenings did you do sports, dance, or play games in which you were very active? (Check one only.)  None .................................................................. օ  1 time last week ................................................ օ  2 or 3 times last week ........................................ օ  4 or 5 last week ................................................ օ  6 or 7 times last week ........................................ օ | 6. V posledních 7 dnech: V kolika dnech ses navečer věnoval nějakému sportu, hraní her nebo jiným pohybovým aktivitám, u kterých jsi byl velmi aktivní (hodně ses zadýchal nebo zpotil)? Navečer se rozumí doba mezi 6 hodinou večer a spánkem. (Označ křížkem pouze jednu odpověď.)  V žádném dni ................................................... օ  V 1 dni .............................................................. օ  Ve 2 nebo 3 dnech ............................................ օ  Ve 4 nebo 5 dnech ............................................ օ  V 6 nebo 7 dnech .............................................. օ |
| 7. On the last weekend, how many times did you do sports, dance, or play games in which you were very active? (Check one only.)  None .................................................................. օ  1 time ................................................................. օ  2 — 3 times ........................................................ օ  4 — 5 times ........................................................ օ  6 or more times …............................................... օ | 7. Během víkendu: Kolikrát ses věnoval nějakému sportu, hraní her nebo jiným pohybovým aktivitám, u kterých jsi byl velmi aktivní (hodně ses zadýchal nebo zpotil)?  (Označ křížkem pouze jednu odpověď.)  Vůbec ............................................................... օ  1krát ................................................................. օ  2 – 3krát ........................................................... օ  4 – 5krát ............................................................ օ  6 a vícekrát ....................................................... օ |

| 8. Which one of the following describes you best for the last 7 days?  Read all five statements before deciding on the one answer that describes you.  A. All or most of my free time was spent doing things that  involve little physical effort .................................................................................. օ  B. I sometimes (1 — 2 times last week) did physical things in my free time  (e.g. played sports, went running, swimming, bike riding, did aerobics) ............ օ  C. I often (3 — 4 times last week) did physical things in my free time .................... օ  D. I quite often (5 — 6 times last week) did physical things in my free time ............ օ  E. I very often (7 or more times last week) did physical things in my free time ....... օ | 8. V posledních 7 dnech: Která z následujících vět nejlépe popisuje, co jsi během posledních 7 dní dělal? Nejdříve si přečti všechny odpovědi. Potom vyber a označ křížkem pouze tu, která Tě nejvíc vystihuje.  A. Všechen nebo většinu svého volného času jsem se věnoval aktivitám,  které vyžadovaly malé fyzické úsilí ...................................................................... օ  B. Občas (1–2krát za poslední týden) jsem se ve svém volném čase věnoval pohybovým aktivitám, u kterých jsem byl hodně zadýchaný a zpocený .............. օ  C. Často (3–4krát) jsem se ve svém volném čase věnoval pohybovým  aktivitám, u kterých jsem byl hodně zadýchaný a zpocený ................................. օ  D. Docela často (5–6krát) jsem se ve svém volném čase věnoval pohybovým aktivitám, u kterých jsem byl hodně zadýchaný a zpocený ................................ օ  E. Velmi často (7 nebo vícekrát) jsem se ve svém volném čase věnoval  pohybovým aktivitám, u kterých jsem byl hodně zadýchaný a zpocený ............. օ |
| --- | --- |
| 9. Mark how often you did physical activity (like playing sports, games, doing dance,  or any other physical activity) for each day last week.   \|  \| None \| Little bit \| Medium \| Often \| Very often \| \| --- \| --- \| --- \| --- \| --- \| --- \| \| Monday ................. \| օ \| օ \| օ \| օ \| օ \| \| Tuesday ................. \| օ \| օ \| օ \| օ \| օ \| \| Wednesday .............. \| օ \| օ \| օ \| օ \| օ \| \| Thursday ................. \| օ \| օ \| օ \| օ \| օ \| \| Friday ...................... \| օ \| օ \| օ \| օ \| օ \| \| Saturday ................... \| օ \| օ \| օ \| օ \| օ \| \| Sunday ..................... \| օ \| օ \| օ \| օ \| օ \| | 9. V posledních 7 dnech: Označ, jak často ses během celého dne věnoval pohybovým aktivitám. Pozor na pořadí dnů v tabulce! Příklad: pokud je dnes čtvrtek, pak se ptáme na minulý čtvrtek až včerejší středu. (V každém řádku označ křížkem pouze jednu odpověď.)   \|  \| nikdy \| občas \| středně často \| často \| Velmi často \| \| --- \| --- \| --- \| --- \| --- \| --- \| \| Pondělí .................... \| օ \| օ \| օ \| օ \| օ \| \| Úterý ...................... \| օ \| օ \| օ \| օ \| օ \| \| Středa ..................... \| օ \| օ \| օ \| օ \| օ \| \| Čtvrtek .................... \| օ \| օ \| օ \| օ \| օ \| \| Pátek ........................ \| օ \| օ \| օ \| օ \| օ \| \| Sobota ..................... \| օ \| օ \| օ \| օ \| օ \| \| Neděle ..................... \| օ \| օ \| օ \| օ \| օ \| |
| 10. Were you sick last week, or did anything prevent you from doing your normal physical activities? (Check one.)    Yes ...................................................……օ  No ............................................................ օ    If Yes, what prevented you? __________________________________ | 10. V posledních 7 dnech: Byl jsi v průběhu posledních 7 dní nemocný nebo ti něco jiného bránilo věnovat se pohybovým aktivitám, kterým se normálně věnuješ?  (Označ křížkem pouze jednu odpověď.)    Ano ...................................................……օ  Napiš, co ti bylo: __________________________________  Ne ............................................................ օ |
