# Supplementary material for: Adaptation and validation of the Physical Activity Questionnaire for Older Children (PAQ-C) among Czech children
Source: PLoS One. 2021 Jan 12;16(1):e0245256. doi: 10.1371/journal.pone.0245256 (PMC7802927; doi:10.1371/journal.pone.0245256)
Supplement: S2 File — (PDF) [file pone.0245256.s003.pdf]

Dotazník pohybové aktivity dětí
(PAQ-C/CZ)

Milá žákyně, milý žáku,
prosíme o vyplnění dotazníku. Dotazník je anonymní. Pokus se zodpovědět všechny otázky upřímně a jak nejlépe dovedeš – je to pro nás velmi důležité. Cílem dotazníku je zjistit úroveň tvé pohybové aktivity v posledních 7 dnech.

PAMATUJ:

1. KŘÍŽKUJ – Své odpovědi označuj křížkem ☒
2. CHYBY – Pokud spleteš odpověď, chybnou zaškrtej ☒ [illegible]vnou nově označ křížkem.
3. POHYBOVÁ AKTIVITA – Jde o různé sporty, tanec, všechny pohybové hry, běžná jízda na kole, chození do školy, se psem, po obchodě, na houbách…, běhání, skákání, lezení, různé práce na zahradě a podobně.
4. 7 DNÍ – V dotazníku se ptáme na pohybové aktivity za posledních 7 dní. Zkus si je vybavit.
5. ŽÁDNÝ TEST – V dotazníku nejsou správné či špatné odpovědi. Nejedná se o žádný test. Nebude to známkováno.

OSOBNÍ ÚDAJE

Pohlaví	☐ dívka	☐ chlapec
Věk	_________ let	
Tělesná výška	_________ cm	
Tělesná hmotnost	_________ kg	

---

1. V posledních 7 dnech: Kterým aktivitám ses věnoval nepřetržitě alespoň půl hodiny? Kolikrát během 7 dní to bylo?
   KROK 1: v celém sloupci označ křížkem, kterým aktivitám ses věnoval (mimo tělocvik).
   KROK 2: označ křížkem, jak často ses svým aktivitám věnoval v posledních 7 dnech.

**KROK 1**	**KROK 2**			
ANO	1–2krát	3–4krát	5–6krát	7krát a více
☐ Atletika (běhání, skákání, házení)	☐	☐	☐	☐
☐ Basketbal	☐	☐	☐	☐
☐ Bojové sporty (judo, karate a jiné)	☐	☐	☐	☐
☐ Florbal	☐	☐	☐	☐
☐ Fotbal	☐	☐	☐	☐
☐ Gymnastika	☐	☐	☐	☐
☐ Házená, vybíjená	☐	☐	☐	☐
☐ In-line bruslení, bruslení na ledě	☐	☐	☐	☐
☐ Jízda na kole, koloběžce (ne e-kolo a e-koloběžka)	☐	☐	☐	☐
☐ Jízda na koni	☐	☐	☐	☐
☐ Lední hokej	☐	☐	☐	☐
☐ Parkour, street workout	☐	☐	☐	☐
☐ Plavání a jiné aktivity ve vodě	☐	☐	☐	☐
☐ Posilovací cvičení, fitness, jóga	☐	☐	☐	☐
☐ Lyžování, běžky, snowboarding	☐	☐	☐	☐
☐ Skateboarding, penny board	☐	☐	☐	☐
☐ Softball, baseball	☐	☐	☐	☐
☐ Tanec, aerobik, hip-hop, street-dance	☐	☐	☐	☐
☐ Tenis, squash, stolní tenis, soft-tenis, badminton	☐	☐	☐	☐
☐ Turistika, delší chůze	☐	☐	☐	☐
☐ Volejbal, beach volejbal	☐	☐	☐	☐
☐ Jiné	☐	☐	☐	☐

2. V posledních 5 školních dnech: V kolika dnech ses *ráno před školou* věnoval nějakému sportu, hraní her nebo jiným pohybovým aktivitám, u kterých jsi byl velmi aktivní (hodně ses u nich zadýchal, zpotil a unavil)?
(Označ křížkem pouze jednu odpověď.)

- ☐ v žádném dni;
- ☐ v 1 dni;
- ☐ ve 2 nebo 3 dnech;
- ☐ ve 4 dnech;
- ☐ v 5 dnech.

3. V posledních 5 školních dnech: Kolikrát jsi byl *v tělocviku* velmi aktivní? Velmi aktivní je intenzivní hraní, běhání, skákání, házení, plavání, u kterého jsi byl hodně zadýchaný a zpocený.
(Označ křížkem pouze jednu odpověď.)

- ☐ neměl jsem tělocvik nebo jsem necvičil;
- ☐ málokdy;
- ☐ občas;
- ☐ docela často;
- ☐ skoro pořád nebo pořád.

4. V posledních 5 školních dnech: Co jsi dělal po většinu času *o všech přestávkách* ve škole? Počítej zde i dobu mezi příchodem do školy a začátkem vyučování.
(Označ křížkem pouze jednu odpověď.)

- ☐ seděl (povídal, četl, plnil školní povinnosti);
- ☐ postával jsem nebo se pomalu procházel;
- ☐ trochu jsem pobíhal nebo si hrál (bez výraznějšího zadýchání);
- ☐ docela hodně jsem pobíhal nebo si hrál (zadýchal jsem se víc, než při běžné chůzi);
- ☐ po většinu času jsem intenzivně běhal nebo si hrál (hodně jsem se zadýchal a zpotil).

5. V posledních 5 školních dnech: V kolika dnech ses *hned po škole a odpoledne* věnoval nějakému sportu, hraní her nebo jiným pohybovým aktivitám, u kterých jsi byl velmi aktivní (hodně ses zadýchal nebo zpotil)?
   Jedná se o dobu mezi odchodem z budovy školy a přibližně 6 hodinou večer.
   (Označ křížkem pouze jednu odpověď.)

   - ☐ v žádném dni;
   - ☐ v 1 dni;
   - ☐ ve 2 nebo 3 dnech;
   - ☐ ve 4 dnech;
   - ☐ v 5 dnech.

6. V posledních 7 dnech: V kolika dnech ses *navečer* věnoval nějakému sportu, hraní her nebo jiným pohybovým aktivitám, u kterých jsi byl velmi aktivní (hodně ses zadýchal nebo zpotil)?
   Navečer se rozumí doba mezi 6 hodinou večer a spánkem.
   (Označ křížkem pouze jednu odpověď.)

   - ☐ v žádném dni;
   - ☐ v 1 dni;
   - ☐ ve 2 nebo 3 dnech;
   - ☐ ve 4 nebo 5 dnech;
   - ☐ v 6 nebo 7 dnech.

7. Během víkendu: Kolikrát ses věnoval nějakému sportu, hraní her nebo jiným pohybovým aktivitám, u kterých jsi byl velmi aktivní (hodně ses zadýchal nebo zpotil)?
   (Označ křížkem pouze jednu odpověď.)

   - ☐ vůbec;
   - ☐ 1krát;
   - ☐ 2 – 3krát;
   - ☐ 4 – 5krát;
   - ☐ 6 a vícekrát.

8. V posledních 7 dnech: Která z následujících vět nejlépe popisuje, co jsi během posledních 7 dní dělal?
Nejdříve si přečti všechny odpovědi. Potom vyber a označ křížkem pouze tu, která Tě nejvíc vystihuje.

- ☐ Všechen nebo většinu svého volného času jsem se věnoval aktivitám, které vyžadovaly malé fyzické úsilí.
- ☐ Občas (1–2krát za poslední týden) jsem se ve svém volném čase věnoval pohybovým aktivitám, u kterých jsem byl hodně zadýchaný a zpocený.
- ☐ Často (3–4krát) jsem se ve svém volném čase věnoval pohybovým aktivitám, u kterých jsem byl hodně zadýchaný a zpocený.
- ☐ Docela často (5–6krát) jsem se ve svém volném čase věnoval pohybovým aktivitám, u kterých jsem byl hodně zadýchaný a zpocený.
- ☐ Velmi často (7 nebo vícekrát) jsem se ve svém volném čase věnoval pohybovým aktivitám, u kterých jsem byl hodně zadýchaný a zpocený.

9. V posledních 7 dnech: Označ, jak často ses během celého dne věnoval pohybovým aktivitám. Pozor na pořadí dnů v tabulce! Příklad: pokud je dnes čtvrtek, pak se ptáme na minulý čtvrtek až včerejší středu.
(V každém řádku označ křížkem pouze jednu odpověď.)

	nikdy	občas	středně často	často	velmi často
Pondělí	☐	☐	☐	☐	☐
Úterý	☐	☐	☐	☐	☐
Středa	☐	☐	☐	☐	☐
Čtvrtek	☐	☐	☐	☐	☐
Pátek	☐	☐	☐	☐	☐
Sobota	☐	☐	☐	☐	☐
Neděle	☐	☐	☐	☐	☐

10. V posledních 7 dnech: Byl jsi v průběhu posledních 7 dní nemocný nebo ti něco jiného bránilo věnovat se pohybovým aktivitám, kterým se normálně věnuješ?
(Označ křížkem pouze jednu odpověď.)

- ☐ ANO
  Napiš, co ti bylo: ....
  ....
- ☐ NE

Nyní se ještě jednou podívej, zda jsi skutečně odpověděl na všechny otázky.

DĚKUJEME ZA VYPLNĚNÍ DOTAZNÍKU.

Vyhodnocení dotazníku PAQ-C/CZ

Výpočet celkového skóre dotazníku

Celkové skóre dotazníku je kompozičním průměrem položek 1 až 9. Před jeho výpočtem je nutné přiřadit jednotlivým odpovědím bodové hodnoty a stanovit dílčí položkové skóre.

Skóre položky 1 (P1)

Každé aktivitě nebo skupině aktivit na jednotlivých řádcích seznamu (celkem 22) přiřaďte jeden až pět bodů takto:

- 1 bod, pokud se dítě aktivitě nevěnovalo (v 1. sloupci není u aktivity křížek), nebo
- 2 až 5 bodů, pokud se dítě aktivitě věnovalo, kde 2 body odpovídají frekvenci „1–2krát“, 3 body frekvenci „3–4krát“, 4 body frekvenci „5–6krát“ a 5 bodů frekvenci „7krát a více“.

Výsledné skóre položky je aritmetickým průměrem ze všech takto přiřazených bodů u všech 22 aktivit seznamu.

V některých případech – dle povahy výzkumu – je žádoucí následná korekce výsledného skóre P1. Podrobnější informace viz poznámky níže.

Skóre položek 2 až 8 (P2 – P8)

Odpovědím označeným křížkem přiřaďte body od 1 do 5 tak, že 1 bod odpovídá vždy první možné odpovědi (první řádek), 2 body druhé možné odpovědi (druhý řádek) atd.

Výsledné skóre u P2 až P8 odpovídá vždy přiřazenému počtu bodů.

Skóre položky 9 (P9)

Odpovědím označeným křížkem u jednotlivých dní týdne přiřaďte v řádku body od 1 do 5, kde 1 bod odpovídá vždy první možné odpovědi („nikdy“), 2 body druhé možné odpovědi („občas“) atd.

Výsledné skóre položky je aritmetickým průměrem ze všech takto přiřazených bodů u jednotlivých dní.

Celkové skóre dotazníku

Celkové skóre je aritmetickým průměrem devíti položkových skóre P1 až P9:

$$Celkové\ skóre = \frac{(P1\ +\ P2\ +\ \ldots +\ P9)}{9}$$

Hodnocení celkového skóre

Celkové skóre dotazníku je hodnota v intervalu od 1 do 5, která označuje celkovou úroveň pohybové aktivity dítěte. Hodnota 1 ukazuje na nízkou, hodnota 5 na vysokou úroveň pohybové aktivity dítěte.

Poznámky

Korekce položkového skóre P1

Ukazuje se, že položkové skóre P1 z hlediska pravděpodobnostního rozložení hodnot neodpovídá rozložením u ostatních položek P2 až P9, na což upozornili Janz et al. (2008) a

navrhli korekci individuálních hodnot P1. Jedná se o korekci, jejímž záměrem je přiřadit první položce dotazníku stejnou váhu, jakou mají v celkovém skóre ostatní položky. Korekce individuálních hodnot je vždy vztažena k určité subpopulaci (souboru), z níž dítě pochází. Tuto skutečnost je nutné zohlednit při interpretaci zjištěné úrovně pohybové aktivity. Korekce položkového skóre P1 ($P1_{korekce}$) u jedince dané subpopulace (souboru) je dána vzorcem

$$P1_{korekce} = \frac{4[P1_{individuální} - 1]}{[P1max_{všichni} - 1] + 1}$$

kde $P1_{individuální}$ je původní hodnota položkového skóre jedince a $P1max_{všichni}$ je maximální hodnota položkového skóre zjištěná v celé subpopulaci (souboru) dětí, do které je jedinec zařazen.

S ohledem na povahu výzkumu je nutné zvážit, zda je žádoucí korekci provést, či zda je korekce bezpředmětná, nebo dokonce nežádoucí.
Pro provedení korekce je nutné, aby byl jedinec součástí početnější kohorty. Při porovnávání podskupin v dané kohortě, typicky například srovnání chlapci vs. dívky, je nutné provést korekci pro každou podskupinu zvlášť.

<u>Korekce doporučujeme</u> provádět zejména v těchto případech:

- deskriptivní studie (popis úrovně pohybové aktivity určité subpopulace, srovnání úrovně pohybové aktivity u různých subpopulací, popis trendů pohybové aktivity určité subpopulace apod.);
- porovnávání úrovně pohybové aktivity určené dle PAQ-C/CZ a jiného nástroje.

<u>Korekce nedoporučujeme</u> provádět zejména v těchto případech:

- popis vztahů mezi pohybovou aktivitou určenou PAQ-C/CZ a jinou proměnnou (korelace);
- experimentální studie (posouzení vlivu intervence na úroveň pohybové aktivity);
- hodnocení intraindividuálních změn;
- komparace s výsledky z jiných studií (tuzemských i zahraničních) neaplikujících korekce P1.

Reference

Janz, K. F., Lutuchy, E. M., Wenthe, P., & Levy, S. M. (2008). Measuring activity in children and adolescents using self-report: PAQ-C and PAQ-A. *Med Sci Sports Exerc., 40*, 767–772. doi: 10.1249/MSS.0b013e3181620ed1
