# Supplementary material for: Adaptation and validation of the Physical Activity Questionnaire for Older Children (PAQ-C) among Czech children
Source: PLoS One. 2021 Jan 12;16(1):e0245256. doi: 10.1371/journal.pone.0245256 (PMC7802927; doi:10.1371/journal.pone.0245256)
Supplement: Attachment — Submitted filename: Czech PAQC.docx [file pone.0245256.s004.docx]

##### PLOS ONE

**Comments to the Authors**

**Ref. No: PONE-D-20-26808**

**Adaptation and validation of the Physical Activity Questionnaire for Older Children among Czech children**

**General Comments:**

Overall, this paper uses strong methodology, which has previously been used in several other validation studies, to validate the PAQ-C in Czech children. The modifications of the questionnaire enable it to be culturally specific and the concurrent validity and intra-class correlations are similar to those from other countries demonstrating that this is an acceptable questionnaire to measure MVPA in this population.

However, I am interested in the generalizability of this population, as I find this the main limitation of the paper. The children were from one area of Czech Republic and only span the ages of 10-13. The proportion of the population that this questionnaire benefits is therefore potentially small. It would be useful to have the authors discuss the socio-economic status of the region and the range of schools in the study to further understand the range of children involved.

The paper would benefit from English editing throughout to correct grammatical mistakes. They are present on all pages of manuscript. For example, on page 3, line 50: the word “because” is not necessary; line 57: requires rewording; line 61: “questionnaires in Czech-language modifications” should be “with modifications”; line 66: “have” should be “has” as you are referring to the PAQ-C.

**Specific Comments:**

Introduction

Page 3, line 46-49: Whilst COVID is at the forefront of the news and current discussions, I do not believe that this paper benefits from referencing the virus. If the authors wish to keep this, I recommend focusing on how older children’s activity patterns may have changed due to various restrictions and not on how promoting PA could strengthen the immune system. However, I would recommend expanding on the first two sentences of the introduction instead.

Page 3, line 51: “movement behaviour” – I do not believe that the word movement is necessary. It could also be moved further up in the sentence “Measuring PA is a challenging task as movement is a complex, multi-dimensional human behaviour”.

Page 3, line 63 -65: A citation is necessary for these claims

Methods

Page 5, line 114: Could the authors please tell the readers more about the schools and location that the test was administered? Is this area and the schools generalizable to the rest of Czech Republic?

Page 5, line 119 - 120: Could the authors please state why the expert board and teachers felt that students under the age of 10 would be unable to complete the questionnaire accurately? Is there a reason that children aged 14 were not invited to participate in the study? My understanding is that the Canadian version was intended for students in grades 4 – 8, who could be between the ages of 8 – 14 years.

Results

Page 7, lines 177 – 179: Are there students that participated in both phases of the study as 169 + 63 takes me above the 223 children stated in line 177. Were the 14 excluded subjects from both phase 1 and 2? If the students that did the accelerometry in phase 1 are different from those who did re-test reliability in phase 2, I think this must be made clear in the methodology and the reasoning explained.

Page 11, line 221: not statistically significant needs to replace statistically non-significant. They imply different things. We cannot claim that it is non-significant, only that it isn’t significant.

Table 1

Phase 2, n does not include the number of girls

Table 2

Phase 2 includes concurrent validity (the 139 students that did both the accelerometry data and PAQ-C). According to the methods section on page 6, this is part of phase 1.

Discussion

Page 11, line 232: How does this questionnaire differ from the other 2 stated questionnaires? Is it shorter? Does it require less time? Is it more or less difficult? Please state the direction as readers may not be familiar with the other two questionnaires.

Page 12, line 251 – 253: Do the authors have any thoughts as to why there was no difference between boys and girls in this population? This was also shown in your accelerometry data for MVPA and could add to the discussion.

Page 15, line 320: I would caution the authors on using the words “identical methods” as it implies all the details are the same, which is not true. For example, the Polish version and the Chinese version used 5s epochs to quantify MVPA by accelerometry, which differs to the 15s used here. I would recommend “followed similar protocols”.

Page 15, line 326 – 332: Here the authors explain why children below 10 were not used in the test, but is there a reason that 14-year-old children were also excluded? Could the authors please explain.

Conclusion

Page 15, line 341: The first 4 words of the sentence do not flow into the rest of the sentence.
